# Supplementary material for: Enhanced Expansion of Human Pluripotent Stem Cells and Somatic Cell Reprogramming Using Defined and Xeno-Free Culture Conditions
Source: Bioengineering (Basel). 2023 Aug 24;10(9):999. doi: 10.3390/bioengineering10090999 (PMC10525589; doi:10.3390/bioengineering10090999)

Supplementary Figure S1

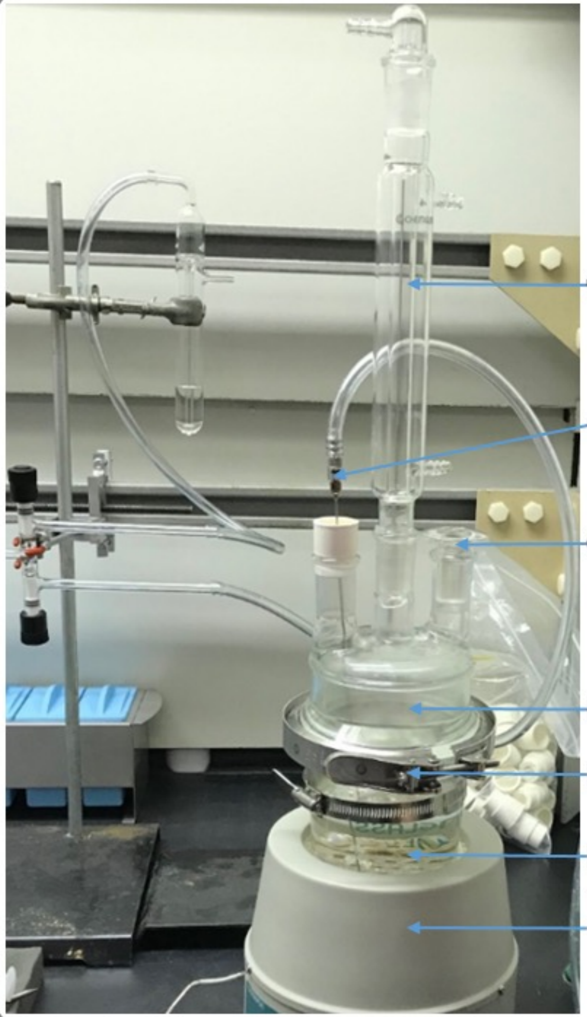

Supplementary Figure S2

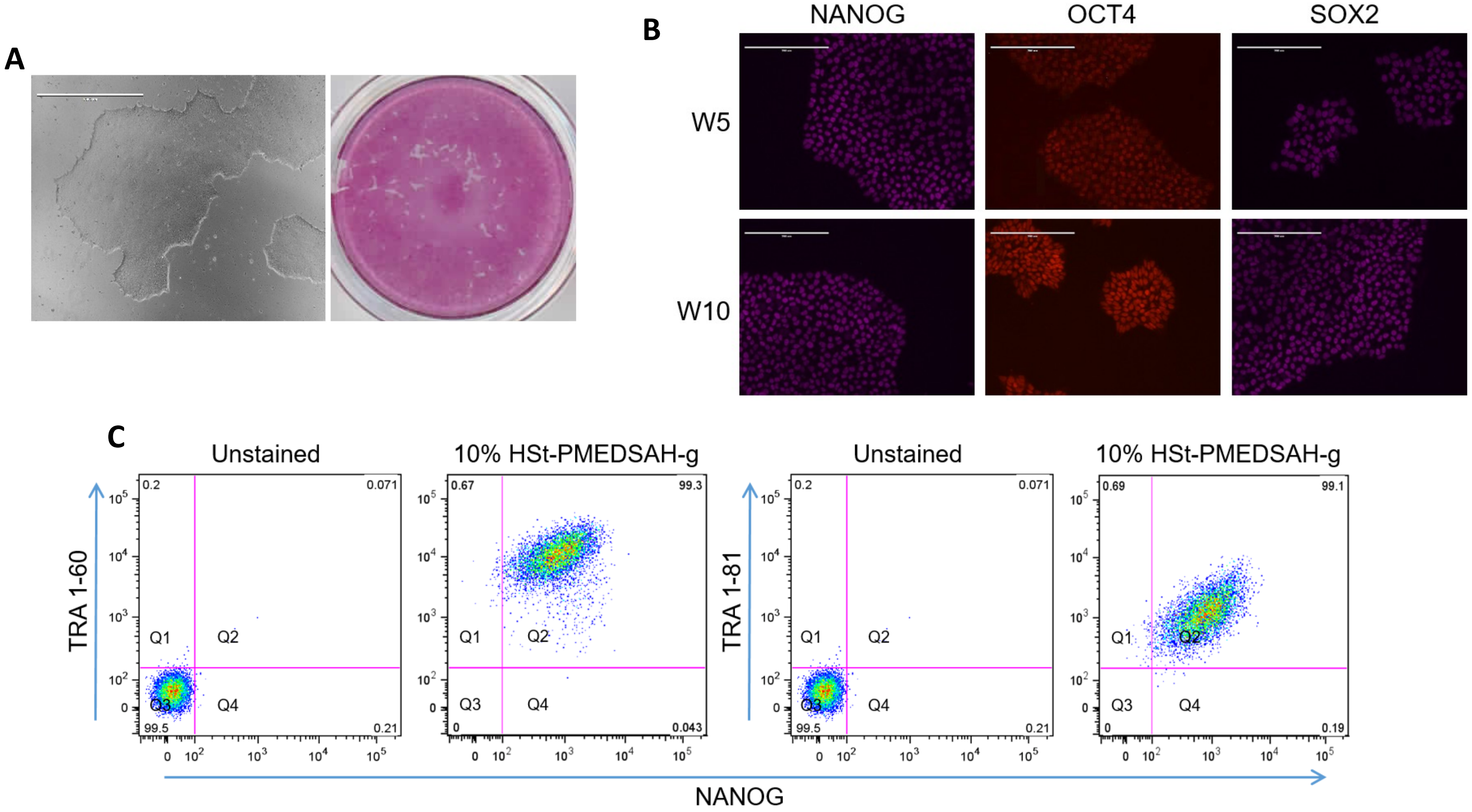

Supplementary Figure S3

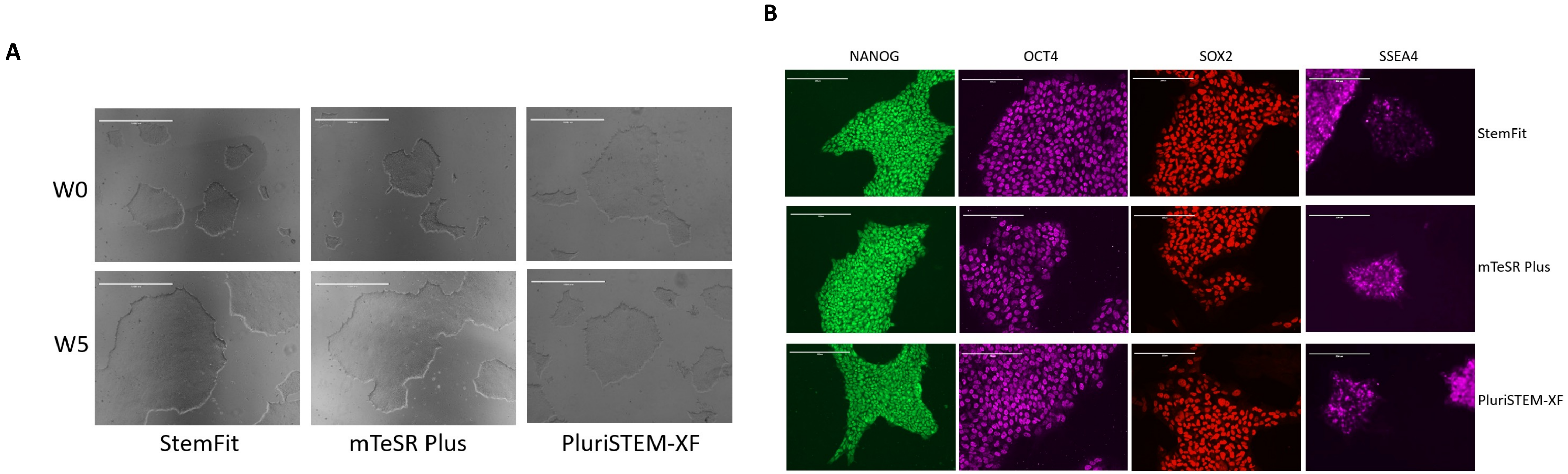

Supplementary Figure S4

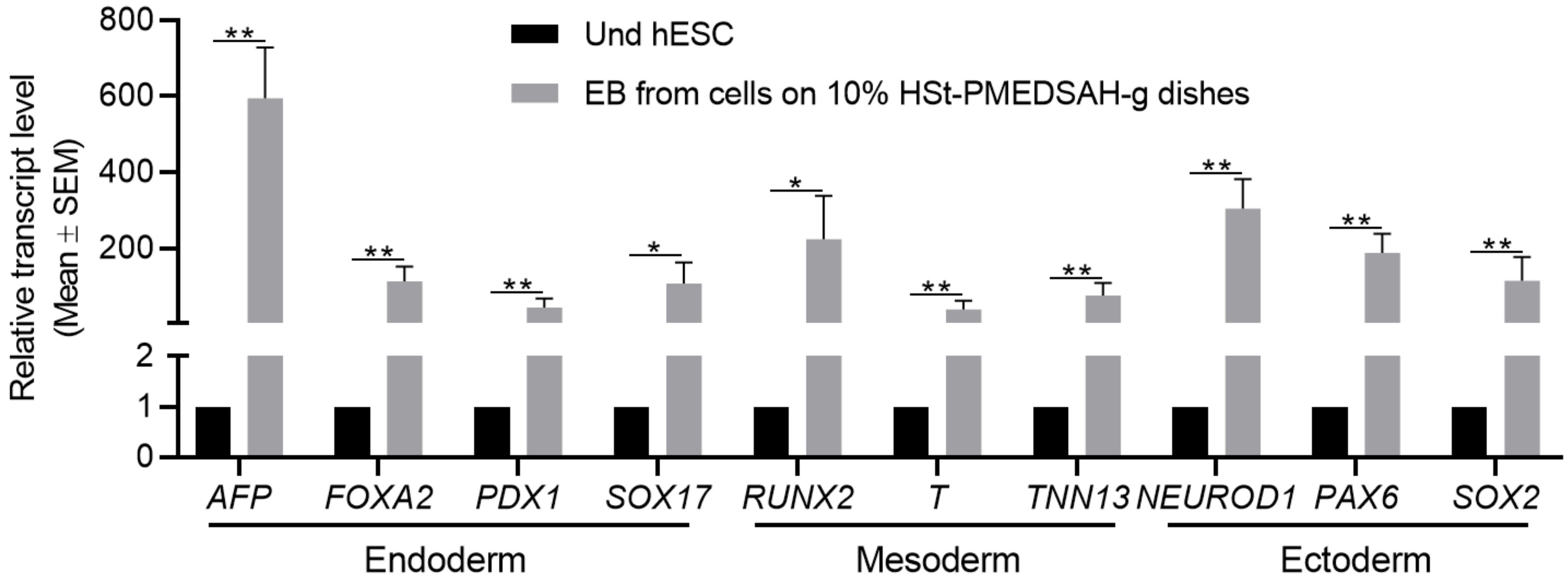

Supplementary Figure S5

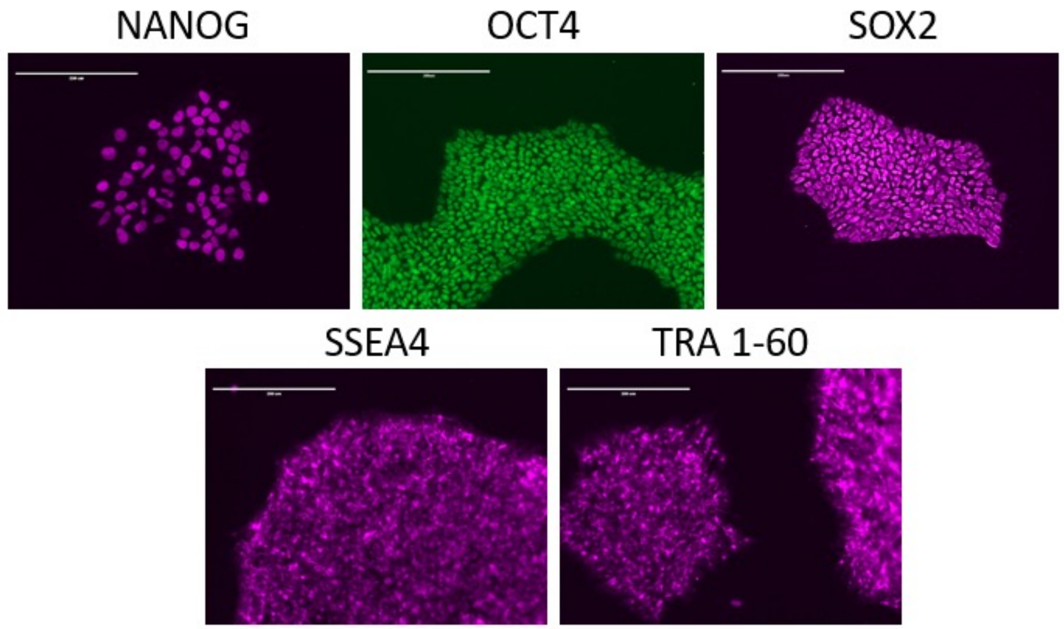

Supplement: Supplementary file 1 [file bioengineering-10-00999-s001.zip › Supplementary figures S1-5.pdf]
