# Supplementary material for: Enhanced Expansion of Human Pluripotent Stem Cells and Somatic Cell Reprogramming Using Defined and Xeno-Free Culture Conditions
Source: Bioengineering (Basel). 2023 Aug 24;10(9):999. doi: 10.3390/bioengineering10090999 (PMC10525589; doi:10.3390/bioengineering10090999)
Supplement: Supplementary file 1 [file bioengineering-10-00999-s001.zip › Supplementary Tables .pdf]

Supplementary Table S1

Medium	Components	XF/CD
StemFlex™	DMEM/F12 bFGF, TGFβ, insulin, transferrin, selenium, ascorbic acid	✓
mTeSR™ Plus	DMEM/F12, with recombinant HSA, bFGF, TGFβ, insulin, transferrin, cholesterol, lipids, pipecolic acid, GABA, β-mercaptoethanol	✓
PluriSTEM™	DMEM/F12, HSA Activin A, TGFβ1, bFGF, lipids, insulin, transferrin, selenium	✓
StemFit®	DMEM/F12, BSA bFGF, TGFβ, Activin, transferrin, LR3-IGF1, HRG1β	✓

Supplementary Table 2

Gene Symbol	Assay ID
AFP	HS00173490_m1
CDX2	Hs01078080_m1
FOXA2	Hs00232764_m1
GAPDH	Hs02786624_g1
GATA4	Hs00171403_m1
NEUROD1	Hs00159598_m1
PAX6	Hs00240871_m1
RUNX2	Hs01047973_m1
SOX2	Hs00602736_s1
T	Hs00610080_m1
PDX1	Hs00236830_m1
SOX17	Hs00751752_m1
TNN13	Hs00165957_m1
FGF4	Hs00908886_m1
GDF3	Hs00214087_m1
HAND1	Hs00153453_m1
NKX2.5	Hs00231763_m1
TBX3	Hs00195612_m1
COL2A1	Hs00116540_m1
MYO3B	Hs00510587_m1
SOX1	Hs00846583_m1

Supplementary Table 3

Gene	Category
ACTB	Controls
ACTB	Controls
ACTB	Controls
ACTB	Controls
CTCF	Controls
EP300	Controls
SMAD1	Controls
CDH9	Ectoderm
COL2A1	Ectoderm
DMBX1	Ectoderm
DRD4	Ectoderm
EN1	Ectoderm
LMX1A	Ectoderm
MAP2	Ectoderm
MYO3B	Ectoderm
NOS2	Ectoderm
NR2F1/NR2F2	Ectoderm
NR2F2	Ectoderm
OLFM3	Ectoderm
PAPLN	Ectoderm
PAX3	Ectoderm
PAX6	Ectoderm
POU4F1	Ectoderm
PRKCA	Ectoderm
SDC2	Ectoderm
SOX1	Ectoderm
TRPM8	Ectoderm
WNT1	Ectoderm
ZBTB16	Ectoderm
AFP	Endoderm
CABP7	Endoderm
CDH20	Endoderm
CLDN1	Endoderm
CPLX2	Endoderm
ELAVL3	Endoderm
EOMES	Endoderm
FOXA1	Endoderm
FOXA2	Endoderm

FOXP2	Endoderm
GATA4	Endoderm
GATA6	Endoderm
HHEX	Endoderm
HMP19	Endoderm
HNF1B	Endoderm
HNF4A	Endoderm
KLF5	Endoderm
LEFTY1	Endoderm
LEFTY2	Endoderm
NODAL	Endoderm
PHOX2B	Endoderm
POU3F3	Endoderm
PRDM1	Endoderm
RXRG	Endoderm
SOX17	Endoderm
SST	Endoderm
FGF4	Mesendoderm
GDF3	Mesendoderm
NPPB	Mesendoderm
NR5A2	Mesendoderm
PTHLH	Mesendoderm
T	Mesendoderm
ABCA4	Mesoderm
ALOX15	Mesoderm
BMP10	Mesoderm
CDH5	Mesoderm
CDX2	Mesoderm
COLEC10	Mesoderm
ESM1	Mesoderm
FCN3	Mesoderm
FOXF1	Mesoderm
HAND1	Mesoderm
HAND2	Mesoderm
HEY1	Mesoderm
HOPX	Mesoderm
IL6ST	Mesoderm
NKX2-5	Mesoderm
ODAM	Mesoderm
PDGFRA	Mesoderm
PLVAP	Mesoderm

RGS4	Mesoderm
SNAI2	Mesoderm
TBX3	Mesoderm
TM4SF1	Mesoderm
CD44	Other
JARID2	Other
MYC	Other
SEV	Other
CXCL5	Self-renewal
DNMT3B	Self-renewal
HESX1	Self-renewal
IDO1	Self-renewal
LCK	Self-renewal
NANOG	Self-renewal
POU5F1	Self-renewal
SOX2	Self-renewal
TRIM22	Self-renewal

Supplementary Table 4

Conditions	Calculated total cell numbers from week 0 to week 5					
hPSC culture on PMEDSAH-g plates with StemFlex medium supplemented for 12 hours with	W0	W1	W2	W3	W4	W5
1% HS	1×10^4	1.1×10^5	-	-	-	-
10% HS	1×10^4	2.7×10^5	1.8×10^4	-	-	-
100% HS	1×10^4	3.1×10^5	-	-	-	-
hPSC culture on PMEDSAH-g plates treated for 30 minutes at room temperature with	W0	W1	W2	W3	W4	W5
1% HS/DMEM/F12 (1% HSt-PMEDSAH-g)	1×10^4	1.1×10^5	2.9×10^5	1.6×10^4	6.1×10^3	-
10% HS/DMEM/F12 (10% HSt-PMEDSAH-g)	1×10^4	4.7×10^5	3.8×10^6	9.9×10^6	5.0×10^7	4.9×10^8
100% HS (100% HSt-PMEDSAH-g)	1×10^4	2.7×10^5	3.2×10^3	-	-	-
hPSC culture on TCPS (as control) treated for 30 minutes at room temperature with	W0	W1	W2	W3	W4	W5
1% HS/DMEM/F12	1×10^4	-	-	-	-	-
10% HS/DMEM/F12	1×10^4	3.7×10^2	-	-	-	-
100% HS/DMEM/F12	1×10^4	-	-	-	-	-
